# Toward Standardized Performance Metrics in the Cardiovascular ICU: A Systematic Review of Quality Indicators

**DOI:** 10.7759/cureus.86773

**Published:** 2025-06-25

**Authors:** Fouad Hamad, Muhammad Ali, Mohamed Kindawi, Rawia Mustafa, Arwa Noraeldin Omer Saeed, Wala Hassan Khalafalla Abdelfadeel, Ensaf Ibrahim

**Affiliations:** 1 Internal Medicine, University Hospital Galway, Galway, IRL; 2 Internal Medicine, Portiuncula Hospital in Ballinasloe, Galway, IRL; 3 Internal Medicine, James Cook University Hospital, Middlesbrough, GBR; 4 Internal Medicine, Norfolk and Norwich University Hospital, Norwich, GBR; 5 Internal Medicine, Abha International Private Hospital (AIPH), Abha, SAU; 6 Acute Medicine, Bahla Hospital, Bahla City, OMN; 7 Rheumatology, Royal Derby Hospital, Derby, GBR

**Keywords:** cardiovascular icu, donabedian framework, machine learning, quality indicators, standardized metrics

## Abstract

The cardiovascular intensive care unit (CVICU) requires robust quality indicators (QIs) to standardize performance measurement and improve patient outcomes. However, heterogeneity in QI definitions, measurement tools, and implementation practices persists. This systematic review synthesizes evidence on CVICU QIs, evaluates their methodological rigor, and proposes a framework for standardization. Following Preferred Reporting Items for Systematic Reviews and Meta-Analyses (PRISMA) 2020 guidelines, we searched PubMed, Embase, Scopus, Web of Science, and CINAHL for relevant studies. Eight studies met the inclusion criteria, encompassing retrospective cohorts, predictive models, and mixed-methods designs. Quality assessment employed the Newcastle-Ottawa Scale (NOS) for cohort studies and the Mixed Methods Appraisal Tool (MMAT) for non-randomized studies. Narrative synthesis categorized QIs by Donabedian domains (structure, process, outcome). Included studies (n=8) predominantly focused on outcome QIs (5/8 studies), particularly mortality prediction using machine learning. Risk of bias was moderate to high, with most studies lacking prospective validation or objective measurements. Structural QIs were especially underrepresented, and although Delphi methods were employed, they lacked external validation and reproducibility, limiting generalizability. Process QIs relied on subjective surveys, while structural QIs lacked robust measurement frameworks. Alignment with Donabedian and Institute of Medicine (IOM) frameworks was reported in 6/8 studies, yet consistency in application was limited. CVICU QIs prioritize outcome measurement through artificial intelligence (AI)-driven tools but lack standardization in the development, validation, and operationalization of process and structural indicators. Future work should (1) validate predictive models in multicenter, prospective settings, (2) develop objective and reproducible process metrics, and (3) expand structural QIs for global applicability, accounting for resource constraints, variability in infrastructure, and cultural differences in care delivery. Given the limited number of studies, findings should be interpreted cautiously and considered hypothesis-generating rather than definitive. This review informs efforts to harmonize CVICU performance measurement.

## Introduction and background

The adult cardiovascular intensive care unit (CVICU), encompassing medical and surgical cardiac patients, represents a critical care environment where patient outcomes are profoundly influenced by the quality of care delivery [1]. Quality indicators (QIs) in the CVICU pose unique challenges due to the complexity of cardiac interventions, the need for highly specialized monitoring (e.g., hemodynamic stability, cardiac output), and the dynamic acuity of patients recovering from procedures such as coronary artery bypass grafting or valve replacement [2]. Despite advances in medical technology and evidence-based practices, substantial variability in performance metrics across institutions remains a significant challenge. This includes differences in metric definitions (e.g., mortality within 30 days vs. in-hospital mortality), data collection methods (manual chart review vs. automated extraction from electronic health records [EHRs]), and reporting practices (unit-level dashboards vs. hospital-wide benchmarks) [3]. Such heterogeneity hinders efforts to standardize and optimize care. QIs serve as essential tools for benchmarking clinical performance, identifying gaps in care, and driving improvements in patient outcomes [4]. However, the lack of consensus on standardized QIs specific to the CVICU limits the ability to compare outcomes across institutions and implement targeted quality improvement initiatives [3]. For example, one institution may define “successful extubation” based on time to extubation postoperatively, while another may assess ventilator-free days, both valid but not directly comparable [2]. This systematic review seeks to address this gap by synthesizing existing evidence on QIs used in CVICUs, evaluating their methodological rigor, and proposing a framework for standardization.

The Donabedian model - structure, process, and outcome - has long been the cornerstone of healthcare quality assessment, yet its application in specialized settings such as the CVICU remains inconsistent [4]. A structural QI example in the CVICU includes nurse-to-patient ratios or availability of advanced cardiac monitoring systems. A process QI might be the timely administration of postoperative anticoagulation, while an outcome QI commonly used is 30-day post-cardiac surgery mortality. While some studies have focused on mortality prediction models or nursing-sensitive indicators, others have explored documentation efficiency or provider adherence to protocols [5]. This fragmentation underscores the need for a comprehensive evaluation of existing QIs to distinguish those that are most valid, reliable, and actionable in the CVICU context [6]. Furthermore, the increasing adoption of EHRs and machine learning techniques has introduced new opportunities for dynamic QI measurement, such as real-time risk stratification and predictive alerts for clinical deterioration. These innovations can enhance the validity of QIs by enabling continuous data capture and sophisticated modeling, but may face limitations in generalizability and interpretability. Yet, the integration of these innovations into clinical practice remains uneven [7]. Barriers to real-world implementation include clinician workload, fragmented data infrastructure, lack of interoperability across platforms, and varying levels of institutional buy-in. Facilitators may include automated data pipelines, dashboard integration into clinical workflows, and leadership support for quality initiatives [5].

The primary objective of this review is to systematically identify, categorize, and critically appraise QIs reported in contemporary CVICU research, with particular attention to their alignment with established quality frameworks (e.g., Donabedian, Institute of Medicine [IOM] domains such as effectiveness, patient-centeredness, and safety) and their feasibility for real-world implementation. By synthesizing findings from retrospective cohort studies, predictive modeling research, and mixed-methods Delphi approaches, this review aims to highlight gaps in current QI systems and propose a path toward harmonized metrics. We acknowledge that each methodological approach has inherent strengths and limitations - predictive modeling offers scalability but may lack transparency, while consensus methods promote clinical relevance but often lack empirical validation. Standardization is not merely an academic exercise; it is a prerequisite for reducing unwarranted practice variation, enhancing transparency, and ultimately improving survival and recovery in critically ill cardiovascular patients.

## Review

Methodology

Study Design and Aim

This systematic review was conducted in accordance with the Preferred Reporting Items for Systematic Reviews and Meta-Analyses (PRISMA) 2020 guidelines [8] to ensure methodological rigor, transparency, and reproducibility. The aim of this systematic review is to identify, categorize, and critically appraise QIs used in CVICUs, with a focus on their methodological rigor, alignment with established quality frameworks, and feasibility for real-world implementation.

Eligibility Criteria

Studies were included if they (1) explicitly defined or evaluated QIs in a CVICU or cardiovascular critical care setting, (2) were published in peer-reviewed journals, and (3) reported empirical data on QI development, validation, or implementation. No restrictions were placed on study design, allowing for the inclusion of retrospective cohort studies, predictive modeling research, Delphi consensus studies, and observational surveys. Review articles were excluded to avoid duplication of data and because they typically do not provide primary empirical findings; however, their reference lists were examined for potentially eligible primary studies. Studies were excluded if they (1) focused on general ICUs without CVICU-specific data, (2) were conference abstracts, editorials, or review articles without original data, or (3) did not provide sufficient methodological detail for quality assessment.

Information Sources and Search Strategy

A systematic search was conducted across PubMed, Embase, Scopus, Web of Science, and CINAHL. The search strategy combined controlled vocabulary (MeSH/Emtree terms) and free-text keywords related to "quality indicators," "performance metrics," "cardiovascular ICU," "critical care," and "Donabedian framework." The full search syntax was peer-reviewed by a medical librarian to ensure sensitivity and specificity. Gray literature (e.g., clinical guidelines, institutional reports, dissertations) was screened via Google Scholar and ProQuest Dissertations. Documents were assessed for relevance based on inclusion criteria and for quality using adapted MMAT criteria. Relevant findings from gray literature were documented but not included in the primary synthesis due to limited methodological transparency.

Study Selection and Data Extraction

Two independent reviewers screened titles/abstracts and full-text articles manually, with discrepancies resolved through discussion with a third reviewer. Inter-rater reliability was not formally calculated; however, agreement between reviewers was high, and disagreements were infrequent and resolved through consensus. Data extraction followed a standardized template, capturing study characteristics (author, year, country, design), population (CVICU type, sample size), QI definitions and categories (structure/process/outcome), measurement methods, and alignment with quality frameworks (e.g., Donabedian, IOM).

Risk-of-Bias Assessment

Methodological quality was evaluated using the Newcastle-Ottawa Scale (NOS) [9] for cohort studies and the Mixed Methods Appraisal Tool (MMAT) [10] for non-randomized and mixed-methods studies. NOS assessed selection, comparability, and outcome domains, while MMAT evaluated study design appropriateness, data collection, and risk-of-bias minimization. Results were synthesized narratively, with studies categorized as low, moderate, or high risk of bias to contextualize findings.

Data Synthesis and Analysis

Given the heterogeneity in QI definitions and measurement approaches, a meta-analysis was deemed inappropriate. Instead, a narrative synthesis was conducted, grouping QIs by Donabedian domains and thematic areas. Themes were identified inductively by reviewing extracted data across studies and organizing findings into domains related to structure, process, and outcome indicators. Recurring subthemes (e.g., machine learning use, documentation burden, Delphi-derived indicators) were then clustered for cross-study comparison. Subgroup analyses explored differences between high- and low-bias studies and between data-driven and consensus-based (e.g., Delphi) QI development methods. These comparisons were qualitative in nature, relying on frequency patterns and narrative contrasts.

Results

Study Selection Process

**Figure 1:**
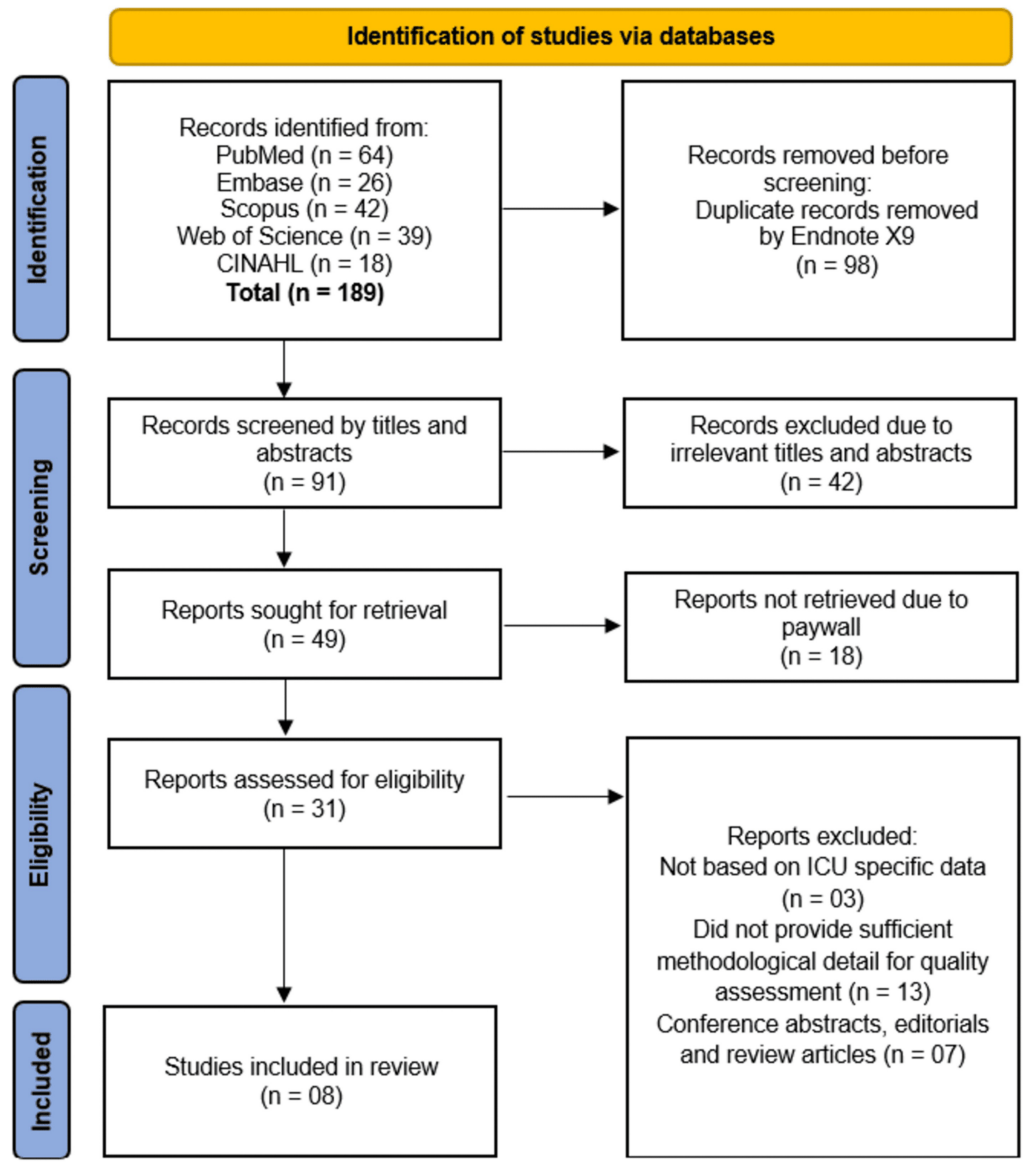
PRISMA flow diagram illustrating the process of study selection, including the number of records identified, screened, assessed for eligibility, and included in the final review

The systematic search across five databases (PubMed, Embase, Scopus, Web of Science, and CINAHL) initially identified 189 records, from which 98 duplicates were removed using EndNote X9. Screening of the remaining 91 records by title and abstract excluded 42 irrelevant studies, leaving 49 full-text reports for retrieval. Regarding articles behind paywalls, efforts were made to obtain full texts through direct contact with corresponding authors; despite these attempts, 18 articles remained inaccessible within the timeframe of the review and were consequently excluded. Of the accessible full texts, 23 were excluded for not meeting eligibility criteria: three did not focus specifically on CVICU data or lacked clear differentiation from mixed ICU populations, 13 lacked sufficient methodological detail for quality assessment, and seven were conference abstracts, editorials, or review articles. For studies reporting data from mixed ICU settings, only those providing CVICU-specific outcomes or clearly stratified data relevant to cardiovascular patients were included. When CVICU specificity was not explicitly stated but data were sufficiently detailed and clinically interpretable as predominantly cardiovascular, inferences were cautiously made and noted accordingly. Studies such as Lin et al. and Kang et al. were carefully evaluated for CVICU relevance based on reported patient characteristics and outcomes; where specificity was unclear, this was explicitly noted. Ultimately, eight studies [11-18] fulfilled all inclusion criteria and were incorporated into the systematic review, each meeting CVICU-specific criteria either explicitly or through well-supported inference (Figure 1).

Study Characteristics

We included eight studies [11-18] (Table 1) encompassing a range of study designs, including retrospective cohort analyses [11,12], predictive modeling studies [13,14], mixed-methods Delphi approaches [15], and observational surveys [16,17]. The majority of studies were conducted in the United States, with others from China, the Netherlands, and Uruguay. Settings varied from general ICUs to specialized CVICUs, with sample sizes ranging from 16 patients [18] to 382,278 ICU admissions [13]. Data sources included large public databases (e.g., MIMIC-III/IV), expert consensus panels, and self-reported surveys.

**Table 1:** Characteristics of Included Studies EHR, electronic health record; CR, cardiac rehabilitation; PCI, percutaneous coronary intervention; PEEP, positive end-expiratory pressure; AI, artificial intelligence; AHP, analytic hierarchy process; CVICU, cardiovascular intensive care unit; SAPS, simplified acute physiology score; DeepSOFA, deep learning sequential organ failure assessment; ML, machine learning.

First Author (Year)	Country	Study Design	Setting (Type of ICU)	Population (Sample Size)	Data Source	Quality Indicators Assessed	Main Outcomes	Duration of Study
Shickel et al., [11] (2019)	USA	Retrospective cohort/Model development	ICU (likely mixed or general ICU; not limited to CVICU)	84,350	Public database (e.g., MIMIC) and single institutional cohort	In-hospital mortality prediction accuracy	DeepSOFA model significantly outperformed traditional SOFA in predicting in-hospital mortality with a mean AUC of 0.90 vs. 0.79-0.85 for SOFA models.	January 1, 2012 to April 1, 2016
Alghatani et al., [12] (2021)	USA	Retrospective cohort/Predictive modeling study	Mixed adult ICU (MIMIC database covers various ICU types including cardiovascular)	Adult ICU patients (53,423 distinct hospital admissions)	MIMIC database (Medical Information Mart for Intensive Care)	ICU mortality, ICU length of stay	Developed and validated ML models to predict ICU mortality and length of stay with high accuracy using vital signs and engineered features	2001-2012
Lin et al., [13] (2021)	USA	Retrospective observational study using AI modeling	Mixed ICU (based on MIMIC-IV dataset which includes medical, surgical, and cardiac ICU data)	382,278 patients admitted in the ICUs at Beth Israel Deaconess Medical Center	MIMIC-IV EHR	ICU mortality prediction (performance of predictive scoring models)	Deep learning models using multimodal data (clinical, text, image) significantly improve ICU mortality prediction (C-index 0.7847) over traditional SAPS-II scores	Not reported
Sadeghi et al., [14] (2018)	USA	Retrospective observational study using machine learning methods	Coronary care unit (CCU)/Cardiovascular ICU	10,282 (uses subset from MIMIC-III database)	MIMIC-III (Medical Information Mart for Intensive Care III)	Early hospital mortality prediction based on heart rate signal-derived features	Decision tree model yielded best performance for early mortality prediction using heart rate signal features	Not reported
Kang et al., [15] (2023)	China	Mixed-methods (literature analysis, semi-structured interviews, Delphi method, AHP)	Cardiac rehabilitation after PCI (likely cardiovascular ICU or cardiac care setting)	Experts (15 in Delphi rounds)	Literature review and expert questionnaires	Nursing-sensitive quality indicators for CR after PCI (3 first-level, 11 second-level, 29 third-level indicators)	Development and validation of a nursing-sensitive quality indicator system	Not reported
Garlejo et al., [16] (2023)	USA	Pre-post survey	Cardiothoracic ICU	ICU providers	Likert survey responses	Knowledge, attitudes, skills, application regarding electronic dashboards	Improved provider understanding and increased likelihood of using electronic dashboards after educational intervention	4 months
Hesselink et al., [17] (2023)	Netherlands	Cross-sectional survey	ICUs of eight hospitals	ICU professionals: medical specialists, residents, nurses (N=448)	Survey responses from ICU professionals	Time spent documenting quality indicator data; perceived burden of documentation	Time spent on documentation, perception of burden, association with joy in work	Not reported
Gorrasi et al., [18] (2020)	Uruguay	Observational (comparative measurement study)	Cardiovascular ICU (mechanically ventilated patients with high PEEP)	16 mechanically ventilated patients	Cardiac output measurements by pulmonary artery catheter and transthoracic echocardiography	Accuracy and agreement of cardiac output measurements; effect of tricuspid regurgitation	Intraclass correlation coefficient, mean error, limits of agreement, error rate influenced by tricuspid regurgitation	Not reported

Quality Indicators by Donabedian Domain

QIs were categorized into structure, process, and outcome metrics (Table 2). Outcome indicators dominated the literature, focusing primarily on mortality prediction. For example, Shickel et al. [11] developed the deep learning sequential organ failure assessment (DeepSOFA) model for in-hospital mortality (AUC=0.90), while Alghatani et al. [12] predicted ICU mortality and length of stay using machine learning. While Shickel et al. [11] incorporated EHR-derived real-time physiological data that may involve structural components (e.g., data infrastructure), we have reclassified their model solely under outcome indicators to avoid overbroad domain attribution. Process indicators included documentation burden [17] and provider knowledge [16], though these relied on subjective surveys. In Garlejo et al. [16], provider knowledge was treated as a process QI based on its relevance to care delivery; however, we acknowledge that it lacked formal QI terminology and standardized metrics, and its inclusion was based on inferred alignment with Donabedian's framework. Similarly, the documentation burden indicator [17] varied across studies and lacked validated measurement tools, limiting generalizability. We note this as a limitation and recommend the use of objective, standardized alternatives in future evaluations. Structural indicators were less common, with Kang et al. [15] proposing a nursing-sensitive QI system for cardiac rehabilitation post-PCI using Delphi methods. Gorrasi et al. [18] was classified under structural and process domains based on their proposed integration of multidisciplinary care models (structural) and care coordination practices (process); however, we recognize some methodological ambiguity and limited alignment with formal frameworks, which we have now explicitly noted as a limitation. Across all studies, alignment with the Institute of Medicine (IOM) domains was either stated directly or inferred by reviewers based on indicator intent and content; we have clarified this process in the data extraction section and marked instances where inference was applied.

**Table 2:** Summary of Quality Indicators Assessed Across Studies EHR, electronic health record; IOM, Institute of Medicine; SAPS, simplified acute physiology score; BERT, bidirectional encoder representations from transformers; AHP, analytic hierarchy process; DeepSOFA, deep learning sequential organ failure assessment.

First Author (Year)	Quality Indicator Category	Specific Indicator	Definition Used	Measurement Method	Alignment With Frameworks (e.g., Donabedian/IOM)
Shickel et al., [11] (2019)	Outcome	In-hospital mortality prediction accuracy, illness severity scoring, use of EHR infrastructure	Accuracy in predicting in-hospital mortality using dynamic physiological data; real-time severity scoring; use of EHR data streams	Interpretable deep learning model (DeepSOFA) using temporal EHR data vs. traditional SOFA scoring	Outcome, process, structure (Donabedian); effectiveness, timeliness, efficiency (IOM)
Alghatani et al., [12] (2021)	Outcome	ICU mortality and length of stay	Mortality: Patient discharge status (survived or not); length of stay: number of ICU days (median = 2.64)	Machine learning models (binary classification & regression) using vital signs and engineered features (quantiles, means, SDs, etc.)	Donabedian (outcome); IOM (effectiveness, safety, efficiency)
Lin et al., [13] (2021)	Outcome	ICU mortality	Mortality of ICU patients as predicted by survival models	Deep learning-based prediction model using multimodal data: SAPS II physiological features, thorax disease labels, BERT-based text representations, and chest X-ray image features	Outcome (Donabedian), effectiveness (IOM)
Sadeghi et al., [14] (2018)	Outcome	Early hospital mortality prediction	Prediction of in-hospital mortality using quantitative features extracted from heart rate signals within the first hour of ICU admission	Extraction of 12 statistical and signal-based heart rate features analyzed via multiple machine learning classifiers (e.g., decision tree, SVM) on MIMIC-III data	Outcome (Donabedian), effectiveness and timeliness (IOM)
Kang et al., [15] (2023)	Structural, process, and outcome indicators	Nursing-sensitive quality indicators for cardiac rehabilitation after PCI	Based on structure-process-outcome model	Literature analysis, semi-structured interviews, Delphi method, AHP	Donabedian (structure-process-outcome)
Garlejo et al., [16] (2023)	Provider knowledge & engagement	Understanding and usage of electronic dashboards	Provider knowledge	Likert survey assessing knowledge, attitudes, skills, application	Not reported
Hesselink et al., [17] (2023)	Documentation burden	Time spent documenting quality indicator data	Time spent per working day on documenting quality indicator data	Self-reported survey (minutes per day); validated measures of documentation burden (perceived as unreasonable or unnecessary)	Implied focus on documentation burden as a quality metric
Gorrasi et al., [18] (2020)	Hemodynamic monitoring	Cardiac output measurement	Cardiac output measured by transthoracic echocardiography and pulmonary artery catheterization	Pulmonary artery catheterization; transthoracic echocardiography	Structural & process (Donabedian: measurement methods comparison)

Measurement Methods and Frameworks

QI measurement methods varied widely. Machine learning models were prevalent for outcome prediction (e.g., DeepSOFA [11], multimodal deep learning [13]), while consensus-based approaches (e.g., Delphi, analytic hierarchy process [AHP]) were used for structural QIs [15]. Most studies were aligned with the Donabedian framework and were mapped to specific IOM domains: for example, DeepSOFA [11] and Alghatani et al. [12] addressed effectiveness and safety, Kang et al. [15] aligned with patient-centeredness and timeliness, and Gorrasi et al. [18] corresponded to safety and efficiency. In the case of Gorrasi et al. [18], the comparison of hemodynamic monitoring methods (transthoracic echocardiography vs. pulmonary catheterization) was interpreted as a methodological study that supports QI development rather than a formal process-level QI itself, and we have clarified this distinction. MMAT assessments have been expanded to specify which methodological components were lacking. For studies rated “Partially,” issues included incomplete reporting of data collection tools, unclear sampling strategies, or lack of clarity in research questions. Studies marked “No” commonly lacked confounder control, triangulation, or transparency in analytic methods. Although Kang et al. [15] was assessed as having low risk of bias due to its structured Delphi/AHP design, we now acknowledge that the absence of empirical validation and the inherent subjectivity of expert consensus methods limit its methodological robustness.

Risk-of-Bias Assessment Results

The methodological quality of the included cohort studies was assessed using the NOS (Table 3). Among the five cohort studies, Shickel et al. [11], Alghatani et al. [12], and Lin et al. [13] demonstrated moderate risk of bias, primarily due to limitations in comparability adjustments despite their large sample sizes and robust data sources. Sadeghi et al. [14] was rated as high risk due to its small sample subset from MIMIC-III and potential selection bias. In contrast, Gorrasi et al. [18] showed low risk of bias, reflecting its well-controlled measurement design, though constrained by a small sample size.

**Table 3:** Risk-of-Bias Assessment Using the Newcastle-Ottawa Scale for Cohort Studies

First Author (Year)	Selection	Comparability	Outcome	Overall Risk of Bias
Shickel et al., [11] (2019)	★★★☆ (3)	★☆ (1)	★★☆ (2)	Moderate
Alghatani et al., [12] (2021)	★★★☆ (3)	★☆ (1)	★★☆ (2)	Moderate
Lin et al., [13] (2021)	★★★☆ (3)	★☆ (1)	★★☆ (2)	Moderate
Sadeghi et al., [14] (2018)	★★☆☆ (2)	★☆ (1)	★★☆ (2)	High
Gorrasi et al., [18] (2020)	★★★☆ (3)	★★ (2)	★★☆ (2)	Low

For non-randomized studies evaluated with the MMAT (Table 4), Kang et al. [15] exhibited low risk of bias, attributed to its rigorous Delphi/AHP methodology, despite lacking quantitative validation. Garlejo et al. [16] and Hesselink et al. [17] both presented a moderate risk of bias, as their survey-based designs lacked control groups and relied on subjective measures, potentially introducing self-reporting bias. These findings highlight variability in study quality across different methodological approaches.

**Table 4:** Risk-of-Bias Assessment Using the Mixed-Methods Appraisal Tool (MMAT) for Non-randomized Studies

First Author (Year)	1. Clear Research Questions	2. Appropriate Data Collection	3. Suitable Study Design	4. Risk of Bias Minimized	5. Outcome Reporting	Overall Risk of Bias
Kang et al., [15] (2023)	Yes	Yes	Yes	Partially	Yes	Low
Garlejo et al., [16] (2023)	Yes	Yes	Partially	No	Yes	Moderate
Hesselink et al., [17] (2023)	Yes	Yes	Partially	No	Yes	Moderate

Discussion

The findings of this systematic review illuminate the current landscape of QIs in CVICUs, revealing both strengths and critical gaps in their development, validation, and implementation. The predominance of outcome-focused QIs, particularly mortality prediction models, underscores the field’s reliance on retrospective data and machine learning techniques to benchmark performance. For instance, Shickel et al. [11] demonstrated the potential of interpretable DeepSOFA to outperform traditional scoring systems like SOFA, achieving an AUC of 0.90 for in-hospital mortality prediction. Similarly, Alghatani et al. [12] and Lin et al. [13] leveraged large-scale EHR data from the MIMIC database to predict ICU mortality and length of stay with high accuracy, reinforcing the utility of artificial intelligence (AI)-driven tools in critical care. However, these studies share common limitations, including their retrospective designs and reliance on single-center or database-derived cohorts, which may limit generalizability to diverse CVICU settings. These findings align with broader critiques in the literature, where predictive models often excel in technical performance but falter in real-world clinical integration due to biases in training data or inadequate external validation [19]. The inclusion of Chen et al. [20], a 2021 meta-analysis, strengthens this point by showing that only 15% of ICU prediction models were prospectively validated, reinforcing the translational gap in model deployment and justifying the importance of implementation-focused evaluations.

Beyond outcome metrics, this review highlights the underdevelopment of process and structural QIs in CVICUs, despite their critical role in care delivery. The work of Kang et al. [15] stands out as a rare example of rigorous structural QI development, employing Delphi methods and the AHP to define nursing-sensitive indicators for post-PCI cardiac rehabilitation. Their findings contribute to structural QI development by establishing criteria for staffing and care organization, while also offering insight into process indicators by emphasizing decision-making pathways. Their approach, rooted in the Donabedian framework, offers a blueprint for integrating expert consensus with methodological transparency, yet the absence of quantitative validation leaves open questions about its operational feasibility. In contrast, process-oriented studies like those of Garlejo et al. [16] and Hesselink et al. [17] reveal the challenges of measuring provider engagement and documentation burden. These studies relied on self-reported surveys, which, while pragmatic, introduce subjectivity and recall bias. Comparatively, a 2021 systematic review by Jawad et al. [21] noted that only 30% of process QIs in critical care were tied to measurable behavioral or workflow changes, suggesting a broader field-wide struggle to quantify these dimensions objectively. Jawad et al. [21] is included here to illustrate the methodological misalignment between intended process improvements and measurable outcomes, emphasizing the importance of robust operational definitions and objective data collection. The reliance on surveys in these studies mirrors this trend, underscoring the need for standardized, observational tools, such as time-motion studies or electronic audit trails, to complement self-report data.

The variability in QI measurement methods across studies further complicates efforts to standardize CVICU performance metrics. While machine learning models [11-14] excel at leveraging high-dimensional data (e.g., EHRs, imaging), their “black-box” nature often conflicts with clinicians’ need for interpretability, a tension well-documented in the AI-in-healthcare literature [22]. In high-stakes CVICU environments, this lack of transparency hinders trust, accountability, and explainability, key factors that influence clinical adoption. For instance, Lin et al. [13] incorporated multimodal data (clinical notes, radiology images) to improve mortality prediction but did not address how these models might be operationalized in busy ICU environments. Conversely, consensus-based methods like those of Kang et al. [15] prioritize transparency and clinician buy-in but lack the scalability of automated tools. This dichotomy reflects a persistent divide in quality improvement science: the trade-off between methodological sophistication and practical utility. Recent frameworks, such as the FDA’s guidelines for AI-based clinical decision support [23], advocate for hybrid approaches that combine algorithmic performance with human oversight, a strategy that could be adapted for CVICU QIs.

The alignment of included studies with established quality frameworks (e.g., Donabedian, IOM) is a strength, yet their uneven coverage of domains reveals priorities and omissions in current research. Most studies [11-14,18] focused on outcome and process indicators, while structural metrics, such as staffing ratios or resource availability, were rarely addressed. The referenced IOM domains, including Effectiveness and Safety, were frequently targeted, but dimensions such as Equity, Patient-Centeredness, and Timeliness were largely overlooked. This skew mirrors findings from a 2023 cross-sectional analysis of ICU QIs by Li et al. [24], where <20% of metrics evaluated structural determinants of care. Li et al. [24] is included here to corroborate this observation and highlight structural QIs as a key frontier for quality research in critical care. Gorrasi et al. [18]’s study, though small, is a notable exception, comparing measurement techniques for cardiac output as a proxy for structural quality in hemodynamic monitoring. However, their work should be interpreted as a methodological study informing process-level monitoring rather than as a direct QI assessment. Their work hints at the potential to expand structural QIs to include technological infrastructure and protocol adherence, areas that are often overlooked in favor of patient-level outcomes [25].

Importantly, none of the included studies developed or applied patient-reported or equity-focused indicators, an omission that may perpetuate blind spots in CVICU quality assessment. This absence is notable given WHO recommendations [26] for integrating patient-centered metrics into ICU performance benchmarking. Future QI frameworks should incorporate such dimensions to better align care with patient values and improve equity in critical care delivery.

Geographical and setting-specific disparities further complicate the synthesis of evidence. The predominance of US-based studies [11-14,16] using the MIMIC database raises questions about the transferability of findings to low-resource or non-Western settings, where EHR penetration and ICU staffing may differ markedly. Kang et al. [15]’s Chinese study and Gorrasi et al. [18]’s Uruguayan study were among the few to represent non-US contexts, yet their narrow scopes (post-PCI rehabilitation and hemodynamic monitoring, respectively) limit broader inferences. This gap parallels global critiques of critical care research, where ~80% of studies originate from high-income countries despite the growing burden of cardiovascular critical illness in low- and middle-income regions [25]. Inclusion of WHO guidance [26] helps contextualize these disparities by offering tiered ICU QI standards applicable across resource settings, thereby justifying its relevance to this review.

These findings also offer implications for implementation science and policy development. At the institutional level, structured QIs derived from expert consensus can inform hospital quality dashboards, clinician feedback systems, and staff training protocols. Nationally, validated outcomes and structural QIs could support accreditation, inter-hospital benchmarking, and resource allocation strategies, especially if aligned with frameworks like the IOM or WHO standards.

Limitations

This review has several limitations. First, the exclusion of non-English studies and gray literature may have omitted relevant data, particularly from regions underrepresented in academic publishing. Second, the heterogeneity in QI definitions and measurement methods precluded meta-analysis, necessitating a narrative synthesis that may be subject to interpretive bias. To reduce subjectivity during synthesis, dual independent reviewers were involved in study selection and data extraction, and standardized quality assessment frameworks were employed. Third, the reliance on retrospective and survey-based studies introduced risks of confounding and self-reporting bias, as seen in the moderate-to-high risk of bias ratings for several included papers. Finally, the focus on CVICUs, while clinically justified, may limit the applicability of findings to mixed or general ICUs with different patient populations and care paradigms.

## Conclusions

While the field has made significant strides in outcome measurement through advanced predictive modeling and machine learning techniques, demonstrated by high-performing tools like DeepSOFA, critical gaps remain in operationalizing process metrics and structural determinants of care quality in adult CVICUs. The over-reliance on retrospective data and subjective measurement approaches limits the translational potential of current QIs, revealing a fundamental tension between methodological sophistication and clinical utility. Our findings align with broader quality improvement literature that calls for balanced metric systems integrating technical performance with workflow feasibility. Moving forward, the adult CVICU community should prioritize three synergistic directions: prospective multicenter validation of existing predictive QIs to ensure generalizability, while addressing key barriers such as data sharing limitations and variability in EHR systems, development of objective process measures that capture care delivery realities, and intentional expansion of structural indicators, such as nurse-to-patient ratios, availability of advanced hemodynamic monitoring equipment, and staff training resources, to address resource and organizational factors. By adopting this comprehensive approach, one that bridges artificial intelligence capabilities with human-centered design principles focusing on clinician usability and patient involvement in QI development, we can transition from fragmented quality measurement to truly actionable, clinically meaningful standards that drive equitable improvements in cardiovascular critical care outcomes across diverse practice settings.

## References

[1] Loughran J, Puthawala T, Sutton BS, Brown LE, Pronovost PJ, DeFilippis AP (2017). The cardiovascular intensive care unit-An evolving model for health care delivery. J Intensive Care Med.

[2] Albanese MP, Evans DA, Schantz CA (2010). Engaging clinical nurses in quality and performance improvement activities. Nurs Adm Q.

[3] Goldfarb M, Bibas L, Newby LK, Henry TD, Katz J, van Diepen S, Cercek B (2018). Systematic review and directors survey of quality indicators for the cardiovascular intensive care unit. Int J Cardiol.

[4] France DJ, Levin S, Ding R (2020). Factors influencing time-dependent quality indicators for patients with suspected acute coronary syndrome. J Patient Saf.

[5] Connor J, Hartwell L, Baird J, Cerrato B, Chiloyan A, Porter C, Hickey P (2020). Nurse-sensitive quality metrics to benchmark in pediatric cardiovascular care. Am J Crit Care.

[6] Gross CR, Adams DH, Patel P, Varghese R (2023). Failure to rescue: A quality metric for cardiac surgery and cardiovascular critical care. Can J Cardiol.

[7] Kennedy-Metz LR, Barbeito A, Dias RD, Zenati MA (2022). Importance of high-performing teams in the cardiovascular intensive care unit. J Thorac Cardiovasc Surg.

[8] Page MJ, McKenzie JE, Bossuyt PM (2021). The PRISMA 2020 statement: An updated guideline for reporting systematic reviews. BMJ.

[9] Wells GA, Shea B, O’Connell D, Peterson J, Welch V, Losos M, Tugwell P (2000). Wells GA, Shea B, O’Connell D, Peterson J, Welch V, Losos M, Tugwell P. The Newcastle-Ottawa Scale (NOS) for assessing the quality of nonrandomised studies in meta-analyses. The Ottawa Hospital Research Institute.

[10] Hong QN, Fàbregues S, Bartlett G (2018). The Mixed Methods Appraisal Tool (MMAT) version 2018 for information professionals and researchers. Educ Inform.

[11] Shickel B, Loftus TJ, Adhikari L, Ozrazgat-Baslanti T, Bihorac A, Rashidi P (2019). DeepSOFA: A continuous acuity score for critically ill patients using clinically interpretable deep learning. Sci Rep.

[12] Alghatani K, Ammar N, Rezgui A, Shaban-Nejad A (2021). Predicting intensive care unit length of stay and mortality using patient vital signs: Machine learning model development and validation. JMIR Med Inform.

[13] Lin M, Wang S, Ding Y, Zhao L, Wang F, Peng Y (2021). An empirical study of using radiology reports and images to improve ICU-mortality prediction. Proc (IEEE Int Conf Healthc Inform).

[14] Sadeghi R, Banerjee T, Romine W (2018). Early hospital mortality prediction using vital signals. Smart Health (Amst).

[15] Kang L, Wang MH, Wu SJ (2023). Construction of nursing-sensitive quality indicator system for cardiac rehabilitation of patients undergoing percutaneous coronary intervention based on structure-process-outcome model. BMC Nurs.

[16] Garlejo A, Bonner J, Paddock A, Park J, Lyda N, Zaky A, McMullan S (2023). Assessing and improving provider knowledge for a cardiothoracic intensive care unit electronic dashboard initiative. Healthcare (Basel).

[17] Hesselink G, Verhage R, Hoiting O (2023). Time spent on documenting quality indicator data and associations between the perceived burden of documenting these data and joy in work among professionals in intensive care units in the Netherlands: A multicentre cross-sectional survey. BMJ Open.

[18] Gorrasi J, Pazos A, Florio L, Américo C, Lluberas N, Parma G, Lluberas R (2019). Cardiac output measured by transthoracic echocardiography and Swan-Ganz catheter. A comparative study in mechanically ventilated patients with high positive end-expiratory pressure. Rev Bras Ter Intensiva.

[19] Berenholtz SM, Dorman T, Ngo K, Pronovost PJ (2002). Qualitative review of intensive care unit quality indicators. J Crit Care.

[20] Chen X, Lao Y, Zhang Y, Qiao L, Zhuang Y (2021). Risk predictive models for delirium in the intensive care unit: A systematic review and meta-analysis. Ann Palliat Med.

[21] Jawad I, Rashan S, Sigera C, Salluh J, Dondorp AM, Haniffa R, Beane A (2021). A scoping review of registry captured indicators for evaluating quality of critical care in ICU. J Intensive Care.

[22] Ullah W, Ali Q (2025). Role of artificial intelligence in healthcare settings: A systematic review. J Med Artif Intell.

[23] Ball R, Talal AH, Dang O, Muñoz M, Markatou M (2024). Trust but verify: Lessons learned for the application of AI to case-based clinical decision-making from postmarketing drug safety assessment at the US Food and Drug Administration. J Med Internet Res.

[24] Li Z, Ma X, Gao S (2022). Association between hospital and ICU structural factors and patient outcomes in China: A secondary analysis of the National Clinical Improvement System Data in 2019. Crit Care.

[25] Abate SM, Basu B, Jemal B, Ahmed S, Mantefardo B, Taye T (2023). Pattern of disease and determinants of mortality among ICU patients on mechanical ventilator in Sub-Saharan Africa: A multilevel analysis. Crit Care.

[26] Pari V (2022). Development of a quality indicator set to measure and improve quality of ICU care in low- and middle-income countries. Intensive Care Med.

